# Immune-metabolic profiling of triple-negative breast cancer during neoadjuvant chemotherapy

**DOI:** 10.1016/j.isci.2026.115845

**Published:** 2026-04-22

**Authors:** Runze Shi, Yuheng Pang, Chen Chen, Kexin Liu, Wenjing Wang, Yuefeng Shang, Zhigao Li, He Ren, Wenzheng Wang

**Affiliations:** 1 Department of Oncological Surgery, Harbin Medical University Cancer Hospital, Harbin, Heilongjiang Province 150000, China; 2 NHC Key Laboratory of Cell Transplantation, The First Affiliated Hospital of Harbin Medical University, Harbin, Heilongjiang Province 150001, China; 3 Genomics Research Center (Key Laboratory of Gut Microbiota and Pharmacogenomics of Heilongjiang Province), College of Pharmacy, Harbin Medical University, Harbin, Heilongjiang Province 150081, China; 4 Beijing Institute of Hepatology, Beijing YouAn Hospital, Capital Medical University, Beijing 100000, P.R. China

**Keywords:** Oncology, Transcriptomics

## Abstract

This multi-omics study investigated immune and metabolic determinants of neoadjuvant chemotherapy (NAC) response in triple-negative breast cancer. Analyzing samples from 32 patients, responders exhibited systemic and intratumoral expansion of metabolically active, functionally competent immune cells, including effector memory T cells and activated dendritic cells with enhanced oxidative phosphorylation. Non-responders displayed immune exhaustion and metabolic suppression, with enriched terminally differentiated T cells and senescent NK cells. Transcriptomic and spatial analyses revealed that non-responder tumors were dominated by hypoxia, driving fatty acid oxidation and lymphocyte dysfunction. These findings demonstrate that pre-treatment immune-metabolic fitness in T and B cells is strongly associated with chemosensitivity, offering a promising avenue for predictive biomarker development.

## Highlights

• Immune-metabolic profiling of TNBC patients during NAC reveals response-related signatures
• Peripheral effector T, DC, and B cell expansion predicts chemosensitivity
• Responders show glycolysis/OXPHOS activation; non-responders show FAO-driven exhaustion
• Hypoxia-induced FAO dependence in non-responders links metabolism to immune dysfunction

## Graphical abstract

**Figure:**
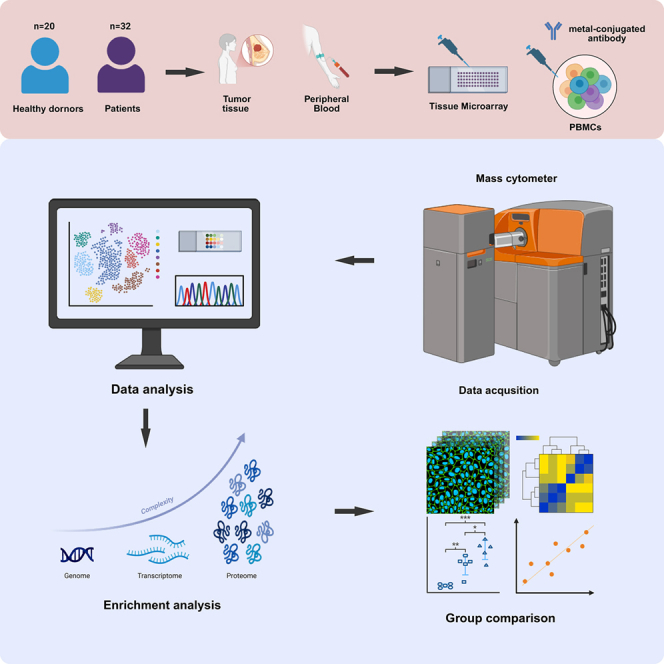

## Introduction

Immune therapy has opened a new perspective in cancer treatment, with its core focus on reversing the suppression of immune cells, modulating, and activating their functions to maximize anti-tumor immune responses.^1^ The importance of immune cells in tumor therapy has been widely recognized, with their functional status playing a decisive role in the prognosis of cancer patients. This predictive value is not only relevant for patients receiving immunotherapy, but is also of significant importance for those undergoing chemotherapy.^2^ Due to the high heterogeneity of tumors, it is difficult to comprehensively assess immune cell infiltration in the tumor microenvironment using biopsy alone. Several studies have now confirmed that the characteristics of tumor-infiltrating immune cells are closely related to the composition of immune cells in peripheral blood.^3^ Analyzing immune cells in peripheral blood provide an indirect evaluation of the immune characteristics of tumors, which can offer valuable insights for predicting immune responses and optimizing treatment strategies.^4^

Immune cells possess complex metabolic patterns, and their functional status is closely linked to metabolic activity.^5^ During neoadjuvant chemotherapy, chemotherapy not only profoundly alters the metabolic environment of tumor cells, but may also influence the metabolic pathways of immune cells, further modulating their functions and anti-tumor effects.^6^

Traditional flow cytometry has limitations in single-cell protein analysis due to the constraints of channel numbers and interference between channels. However, mass cytometry (CyTOF), a breakthrough derivative of flow cytometry technology, replaces fluorescence markers with metal isotopes, significantly increasing the number of detectable channels while reducing channel interference.^7^ This high-throughput proteomic technique provides a novel approach to tumor immunology, enabling the simultaneous evaluation of multiple immune cells at the single-cell level.^8^

In this study, we employed CyTOF technology to detect multiple different protein markers and comprehensively analyzed the abundance, metabolic patterns, and functional status of immune cells and their subpopulations in the peripheral blood and tumor of several triple-negative breast cancer (TNBC) patients undergoing neoadjuvant chemotherapy. Our findings not only reveal the dynamic changes in immune cells during chemotherapy in triple negative breast cancer patients but also provide new insights into predicting treatment responses and improving therapeutic efficacy. By delving deeper into these changes, we hope to contribute to the development of more precise and personalized treatment strategies for breast cancer patients in the future.

## Results

### Phenotypic changes of PBMCs in triple negative breast cancer patients induced by neoadjuvant chemotherapy

**Figure 1:**
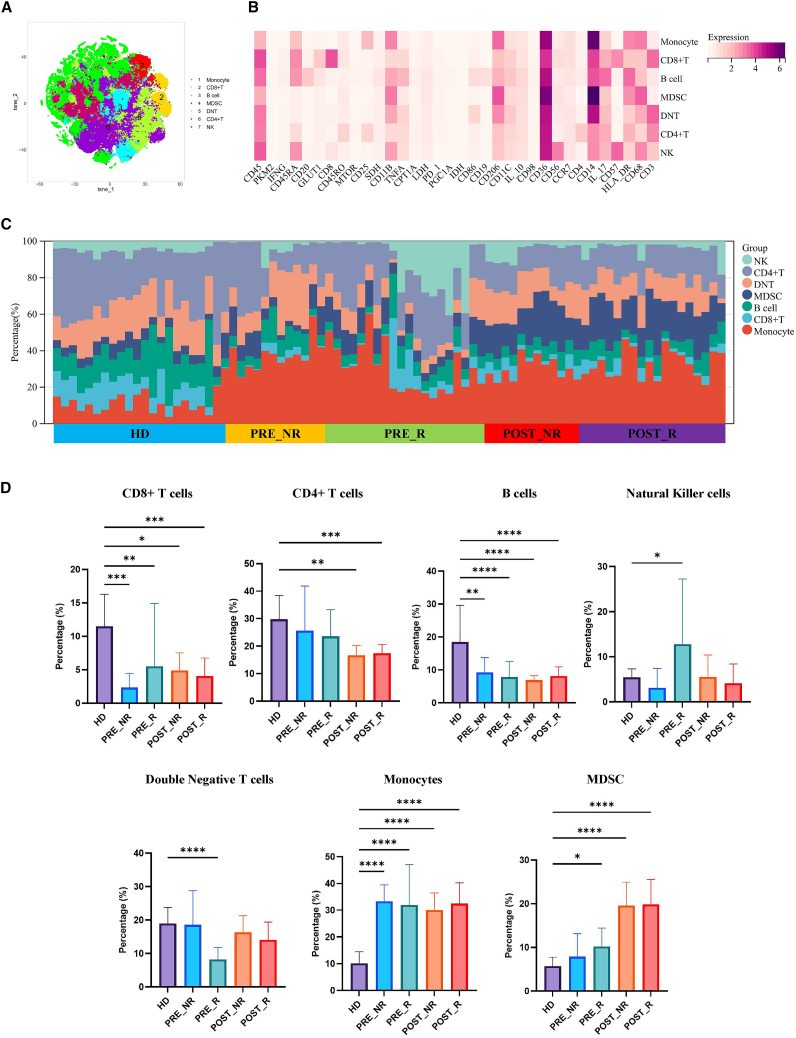
PBMC immune composition differences pre- and post- neoadjuvant chemotherapy in TNBC patients based on treatment response (A) t-SNE plot showing cell clustering subpopulations. (B) Heatmap displaying the relative expression levels of cell proteins. Color bars range from purple to yellow, with yellow indicating higher expression levels. (C) Stacked bar chart showing the proportions of each cell subpopulation in different samples. (D) Boxplot comparing changes in cell subpopulation proportions before and after treatment (each point represents an individual sample, with the bold solid line indicating the mean value). Statistical significance was determined by two-sided Wilcoxon rank-sum test with Benjamini-Hochberg FDR correction. Data are presented as mean ± SEM. *n* = 20 responders, 12 non-responders. (∗*p* < 0.05, ∗∗*p* < 0.01, ∗∗∗*p* < 0.001, ∗∗∗∗*p* < 0.0001).

Peripheral blood samples were collected from female healthy donors and triple negative breast cancer patients undergoing neoadjuvant chemotherapy, one week before and one week after treatment. PBMCs were isolated from the whole blood, and cell clumping and debris were removed in Cytobank. Immune cells were separated based on CD45 expression. Phenograph clustering was performed in R, and dimensionality reduction and visualization were conducted using t-SNE, identifying seven distinct cell subpopulations (Figure 1A). The cells were defined based on the expression profiles of classic cell surface markers (Figure 1B). We compared the proportions of each immune cell population and observed substantial heterogeneity in immune cell distribution across samples (Figure 1C).

Inter-group comparisons revealed that the most pronounced differences occurred between healthy donors and patients, as well as between pre-treatment and post-treatment samples. Based on treatment responses, patients were further stratified into non-responders (NRs) and responders (Rs). Compared with healthy donors, patients with TNBC exhibited markedly reduced proportions of CD8^+^ T cells and B cells in peripheral blood mononuclear cells (PBMCs). Notably, these immune cell populations failed to recover in the short term following neoadjuvant chemotherapy. In addition, the proportion of CD4^+^ T cells further declined after treatment. Prior to treatment, Rs displayed a higher proportion of natural killer (NK) cells and a lower proportion of double-negative T (DNT) cells compared with NRs. However, these differences were no longer observed after neoadjuvant chemotherapy. Furthermore, patients exhibited increased proportions of monocytes and myeloid-derived suppressor cells (MDSCs) relative to healthy donors, with a further elevation of MDSCs observed in the short term following chemotherapy (Figure 1D).

Collectively, these results demonstrate that neoadjuvant chemotherapy induces profound and rapid alterations in the peripheral immune landscape of TNBC patients, characterized by sustained lymphocyte depletion and expansion of immunosuppressive myeloid populations, with distinct baseline immune features associated with treatment response.

### Metabolic characteristics and functional molecule expression changes of CD4^+^ T cells in response to neoadjuvant chemotherapy

**Figure 2:**
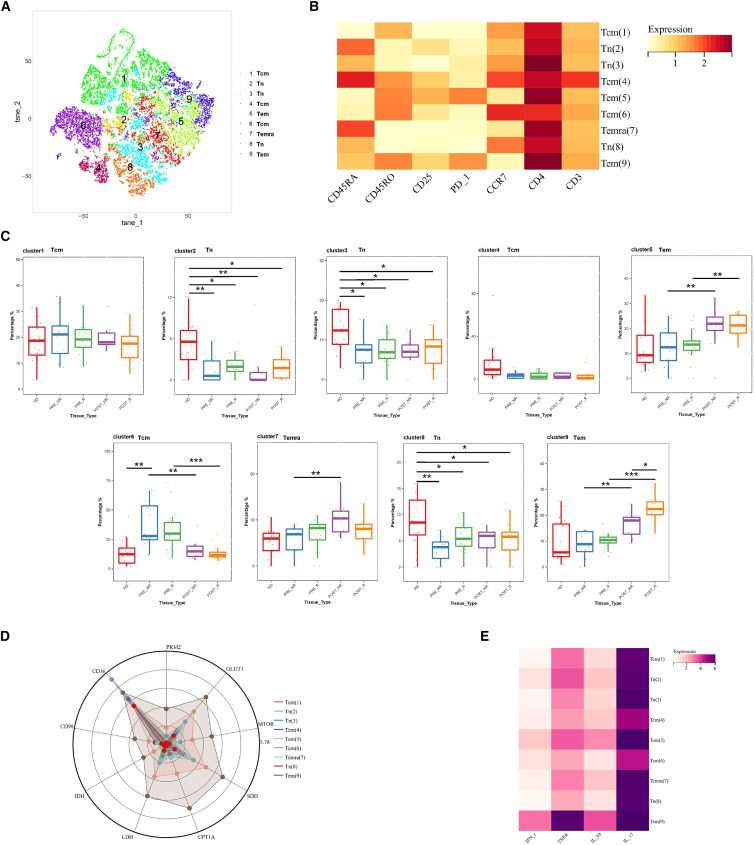
Subpopulation analysis of CD4^+^ T cells (A) t-SNE plot showing cell clustering subpopulations. (B) Heatmap displaying the relative expression levels of surface proteins in each subpopulation. Color bars range from yellow to red, with red indicating higher expression levels. (C) Boxplot comparing the changes in cell subpopulation proportions before and after treatment. (D) Radar plot displaying the relative expression levels of metabolism-related proteins in each cell subpopulation. (E) Heatmap showing the relative expression levels of functional proteins in each subpopulation. Color bars range from white to purple, with purple indicating higher expression levels. Statistical significance was determined by two-sided Wilcoxon rank-sum test with Benjamini-Hochberg FDR correction. Data are presented as mean ± SEM. *n* = 20 responders, 12 non-responders. (∗*p* < 0.05, ∗∗*p* < 0.01, ∗∗∗*p* < 0.001).

To investigate differences in CD4^+^ T cell populations among patients with distinct responses to neoadjuvant chemotherapy, CD4^+^ T cells were isolated and subjected to FlowSOM clustering analysis. A total of nine distinct subpopulations were identified (Figure 2A). Based on the expression profiles of CD45RA, CD45RO, and CCR7, these subpopulations were annotated as three subsets of naive T cells (Tn; clusters 2, 3, and 8), three subsets of central memory T cells (Tcm; clusters 1, 4, and 6), two subsets of effector memory T cells (Tem; clusters 5 and 9), and one subset of terminally differentiated effector memory T cells (Temra; cluster 7) (Figure 2B). Comparison of CD4^+^ T cell subset abundance revealed that Tn cells were predominantly enriched in healthy donors. In contrast, cancer patients exhibited a higher proportion of Tcm cells prior to treatment. Following neoadjuvant chemotherapy, the proportion of Tcm cells decreased, accompanied by a marked expansion of Tem cells. Notably, one effector memory T cell subset (cluster 9) showed a pronounced increase after treatment in the R group. In the NR group, Temra cells were significantly elevated after treatment, consistent with sustained antigen exposure and features associated with T cell exhaustion (Figure 2C).

Analysis of metabolic and molecular expression profiles revealed that effector memory T cell subsets (clusters 5 and 9) exhibited high expression levels of multiple metabolic markers, accompanied by relatively strong expression of IFN-γ, TNF-α, and IL-10. In contrast, one central memory T cell subset (cluster 6) displayed the highest expression of CD36, while the expression of IL-17 and TNF-α remained comparatively low, consistent with enhanced self-renewal capacity and an incompletely activated state of Tcm cells (Figures 2D and 2E). Overall, distinct CD4^+^ T cell subsets exhibited clear functional divergence in metabolic features and effector molecule expression, with Tem cells demonstrating higher metabolic activity and effector potential.

Collectively, CD4^+^ T cell subsets exhibited distinct distribution and functional profiles across disease states and treatment responses. Neoadjuvant chemotherapy was associated with expansion of effector memory T cells, whereas terminally differentiated effector memory T cells were preferentially enriched in NRs. Effector memory T cells displayed the highest metabolic and cytokine expression among CD4^+^ T cell subsets.

### Metabolic characteristics and functional molecule expression changes of B cells in response to neoadjuvant chemotherapy

**Figure 3:**
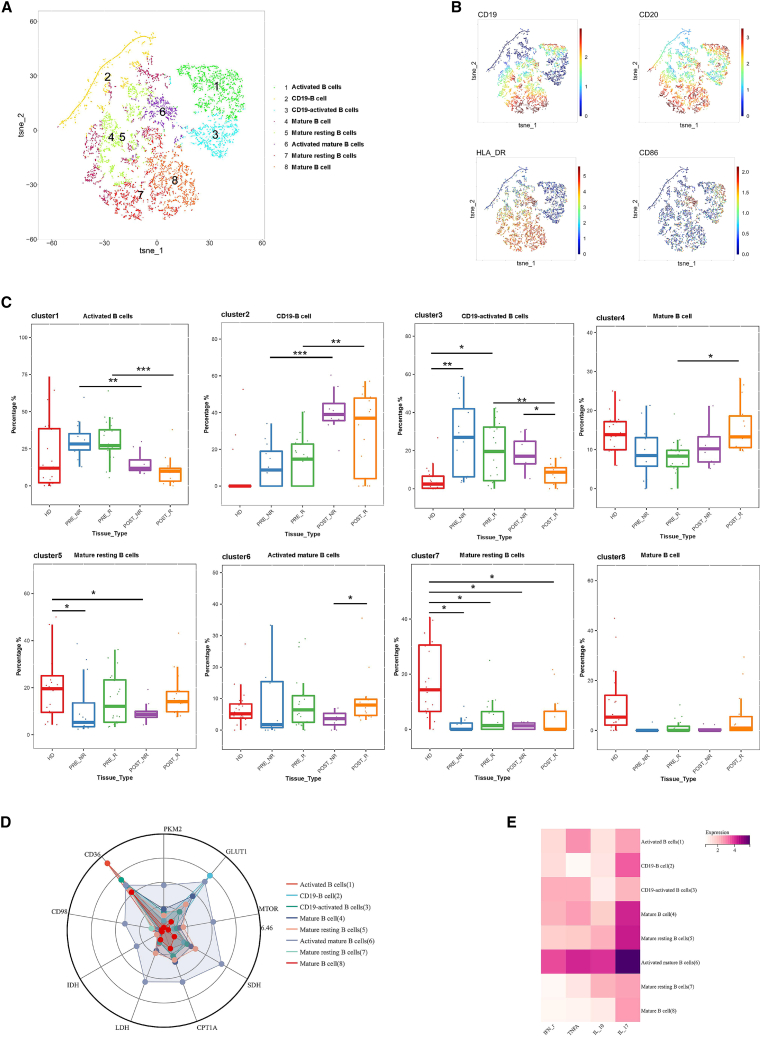
Subpopulation analysis of B cells (A) t-SNE plot showing cell clustering subpopulations. (B) t-SNE heatmap showing the expression levels of CD19, CD20, HLA-DR, and CD86. (C) Boxplot comparing the changes in cell subpopulation proportions before and after treatment. (D) Radar plot displaying the relative expression levels of metabolism-related proteins in each cell subpopulation. (E) Heatmap showing the relative expression levels of functional proteins in each subpopulation. Color bars range from white to purple, with purple indicating higher expression levels. Statistical significance was determined by two-sided Wilcoxon rank-sum test with Benjamini-Hochberg FDR correction. Data are presented as mean ± SEM. *n* = 20 responders, 12 non-responders. (∗*p* < 0.05, ∗∗*p* < 0.01, ∗∗∗*p* < 0.001).

To investigate differences in B cell populations among patients with distinct responses to neoadjuvant chemotherapy, B cells were isolated and subjected to FlowSOM clustering analysis. A total of eight distinct subpopulations were identified (Figure 3A) and annotated based on CD19, CD20, and HLA-DR expression as follows: activated B cells (cluster 1), CD19^−^ B cells (cluster 2), CD19^−^ activated B cells (cluster 3), mature B cells (cluster 4), mature resting B cells (cluster 5), activated mature B cells (cluster 6), mature resting B cells (cluster 7), and mature B cells (cluster 8) (Figure 3B).

Comparison of B cell subset abundance revealed dynamic changes following neoadjuvant chemotherapy. After treatment, Activated B cells and CD19^−^ activated B cells markedly decreased, with CD19^−^ B cells being nearly absent in healthy donors. In contrast, CD19^−^ B cells increased after treatment, while one Mature B cell (cluster 4) subset increased specifically in the R group. Activated mature B cells were more abundant in Rs than in NRs following chemotherapy. Mature resting B cells and some mature B cell (cluster 6) subsets were predominantly enriched in healthy individuals (Figure 3C).

Analysis of metabolic and molecular expression profiles revealed marked heterogeneity across B cell subsets (Figures 3D and 3E). Among all populations, activated mature B cells exhibited the most metabolically active phenotype, characterized by elevated expression of multiple metabolic markers. Importantly, this heightened metabolic activity was accompanied by increased expression of effector and inflammatory cytokines, including IFN-γ, TNF-α, and IL-17, as well as the immunoregulatory cytokine IL-10, indicating enhanced functional capacity. In comparison, activated but relatively immature B cells predominantly expressed CD36, a feature consistent with a lipid uptake-associated metabolic program commonly observed in activated immune cells. By contrast, CD19^−^ B cells showed the highest expression of GLUT1, suggesting a preference for glucose metabolism, and displayed the lowest levels of the pro-inflammatory cytokine TNF-α as well as reduced IL-10 expression, indicative of a less functionally engaged state.

Overall, neoadjuvant chemotherapy reshaped both the composition and functional states of B cell subsets, characterized by R-associated enrichment of metabolically active and cytokine-producing activated mature B cells. In contrast, CD19^−^ and immature B cell subsets exhibited distinct metabolic profiles but limited inflammatory activity, highlighting functional heterogeneity within the B cell compartment following treatment.

### Metabolic characteristics and functional molecule expression changes of CD8^+^ T cells in response to neoadjuvant chemotherapy

**Figure 4:**
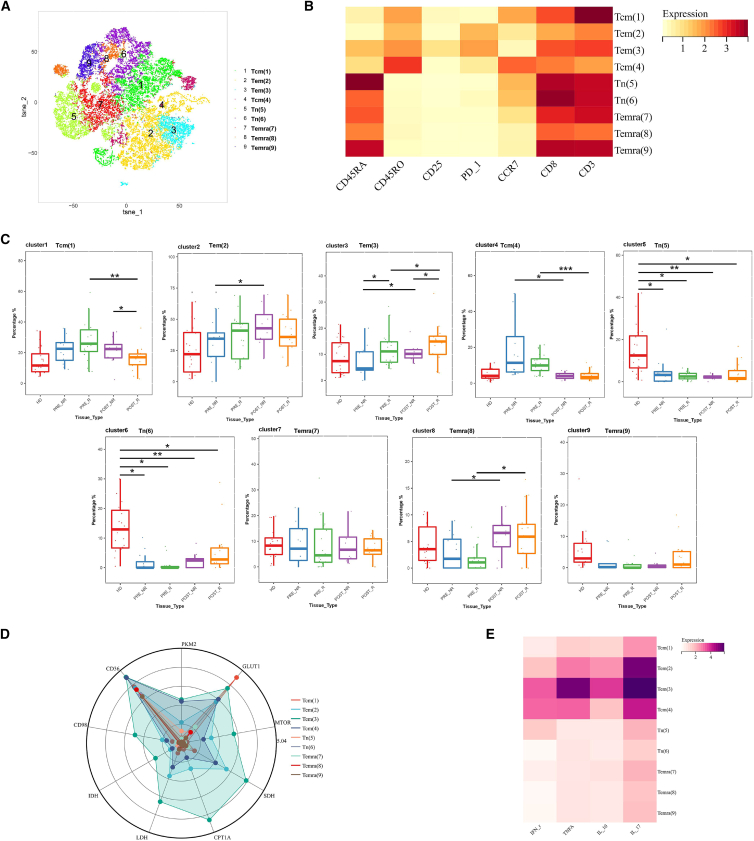
Subpopulation analysis of CD8^+^ T cells (A) t-SNE plot showing cell clustering subpopulations. (B) Heatmap displaying the relative expression levels of surface proteins in each subpopulation. Color bars range from yellow to red, with red indicating higher expression levels. (C) Boxplot comparing the changes in cell subpopulation proportions before and after treatment. (D) Radar plot displaying the relative expression levels of metabolism-related proteins in each cell subpopulation. (E) Heatmap showing the relative expression levels of functional proteins in each subpopulation. Color bars range from white to purple, with purple indicating higher expression levels. Statistical significance was determined by two-sided Wilcoxon rank-sum test with Benjamini-Hochberg FDR correction. Data are presented as mean ± SEM. *n* = 20 responders, 12 non-responders. (∗*p* < 0.05, ∗∗*p* < 0.01, ∗∗∗*p* < 0.001).

To investigate differences in CD8^+^ T cell populations among patients with distinct responses to neoadjuvant chemotherapy, CD8^+^ T cells were isolated and subjected to FlowSOM clustering analysis. A total of nine distinct subpopulations were identified (Figure 4A). Based on the expression profiles of CD45RA, CD45RO, and CCR7, these subpopulations were annotated as two subsets of naive T cells (Tn; clusters 5 and 6), two subsets of central memory T cells (Tcm; clusters 1 and 4), two subsets of effector memory T cells (Tem; clusters 2 and 3), and three subsets of terminally differentiated effector memory T cells (Temra; clusters 7, 8, and 9) (Figure 4B).

Comparison of CD8^+^ T cell subset abundance revealed marked changes associated with neoadjuvant chemotherapy. Naive T cells were predominantly enriched in healthy donors, whereas central memory T cells were more abundant in patients prior to treatment. Following chemotherapy, effector memory T cells showed a marked expansion in patient samples. Notably, one terminally differentiated effector memory T cell subset (cluster 8) increased significantly after treatment (Figure 4C).

Analysis of metabolic and cytokine expression profiles further distinguished CD8^+^ T cell subsets. Effector memory T cell subsets and one central memory T cell subset (cluster 4) exhibited high expression levels of multiple metabolic markers. Among all subsets, one central memory T cell subset (cluster 1) displayed the highest expression of GLUT1. In terms of cytokine expression, one effector memory T cell subset (cluster 3) exhibited the strongest expression of TNF-α, IL-10, and IL-17, whereas one central memory T cell subset (cluster 4) showed relatively high IFN-γ expression (Figures 4D and 4E).

Collectively, CD8^+^ T cell subsets displayed distinct distribution and metabolic profiles across disease states and treatment responses. Neoadjuvant chemotherapy was associated with expansion of effector memory and terminally differentiated effector memory T cells. Effector memory and central memory T cells showed the highest metabolic and cytokine expression among CD8^+^ T cell subsets.

### Transcriptomic profiling reveals enhanced intratumoral immune activation signatures in chemotherapy responders

**Figure 5:**
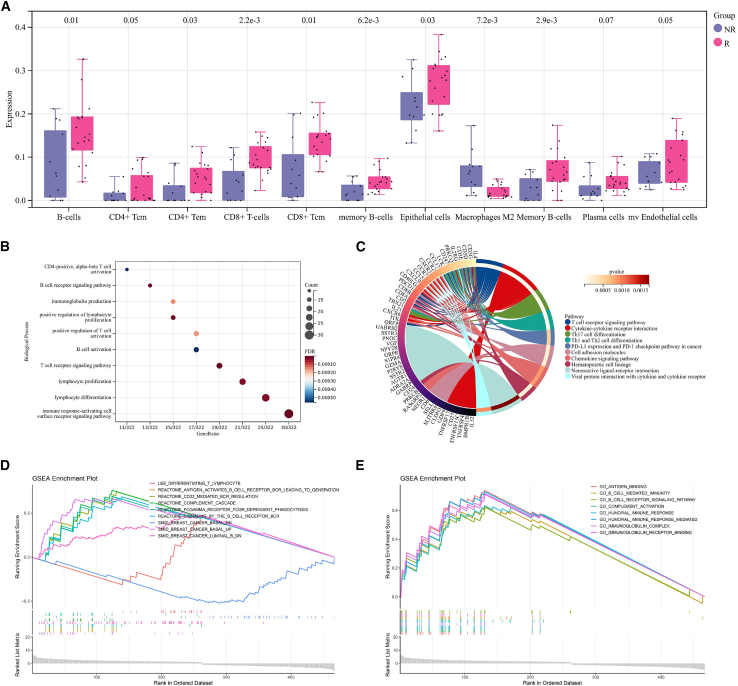
Transcriptomic analysis of tumor tissues from responder and non-responder patients (A) Comparison of tumor-infiltrating immune cell proportions between NR and R groups, estimated by xCell analysis. (B) Gene ontology (GO) enrichment analysis showing the main biological processes affected by differentially expressed genes (DEGs) between NR and R tumors. (C) Kyoto Encyclopedia of Genes and Genomes (KEGG) pathway analysis illustrating signaling pathways perturbed by DEGs. (D) Gene Set Enrichment Analysis (GSEA) based on the C2 (curated gene sets) collection showing transcriptional differences between NR and R groups. (E) GSEA based on the C5 (Gene Ontology) collection revealing enriched biological processes between NR and R groups. Statistical significance was determined by two-sided Wilcoxon rank-sum test with Benjamini-Hochberg FDR correction. *n* = 20 responders, 12 non-responders.

To further explore the potential association between peripheral immune profiles and the tumor immune microenvironment in patients receiving neoadjuvant chemotherapy for TNBC, we performed transcriptomic sequencing on tumor tissues from the same cohort. Cell composition analysis using xCell revealed that, although R tumors contained a higher proportion of malignant epithelial cells, the abundance of B cells (including memory B cells), CD4^+^ central memory T (CD4 TCM) cells, and CD8^+^ T cells (particularly CD8 TCM) was significantly higher in the R group compared with the NR group, while M2 macrophages were reduced (Figure 5A). These findings suggest that differential immune cell infiltration may contribute to the distinct therapeutic responses observed between groups. To elucidate the molecular basis underlying these differences, we identified differentially expressed genes (DEGs) between R and NR tumors and performed GO, KEGG, and GSEA analyses. The results showed that the upregulated biological processes in the R group were primarily associated with CD4^+^ αβ T cell activation, B cell receptor signaling, immunoglobulin production, positive regulation of lymphocyte proliferation and T cell activation, and immune response-activating cell surface receptor signaling (Figure 5B). KEGG pathway enrichment revealed that the most significantly enriched pathways included T cell receptor signaling, cytokine-cytokine receptor interaction, Th17, Th1, and Th2 cell differentiation, PD-L1/PD-1 checkpoint signaling, cell adhesion molecules, chemokine signaling, and hematopoietic cell lineage pathways (Figure 5C). GSEA using the MSigDB C2 (curated gene sets) and C5 (Gene Ontology) collections further supported these findings. In the C2 dataset, pathways related to B cell receptor signaling, T cell differentiation, antibody-mediated immune responses, and myeloid phagocytic activity were significantly enriched in the R group, whereas basal-like breast cancer-downregulated gene sets were more enriched in the NR group (Figure 5D). Within the C5 ontology sets, biological processes such as antigen binding, immunoglobulin receptor binding, humoral immune response, and complement activation were positively enriched in the R group (Figure 5E).

Collectively, both C2 and C5 results indicate that immune activation-related pathways predominate in the R group, while cell proliferation and metabolic pathways are enhanced in NR tumors. Notably, the gene expression profile of R tumors was significantly enriched in the “basal-like breast cancer up” signature. Given that most TNBCs are classified within the basal-like molecular subtype, this finding reinforces the subtype-specific context of our cohort. This suggests that chemosensitivity is closely associated with a basal-like molecular program characterized by higher immunogenic potential, which may facilitate the activation of antitumor immune responses. The pronounced humoral immune activation, reflected by the recruitment and proliferation of B cells in tumors, was consistent with our PBMC findings, where activated B cells and effector memory T cells were expanded in Rs. Together, these observations highlight a peripheral-intratumoral immune activation axis that may serve as a composite biomarker predicting chemotherapy response in breast cancer.

### Spatial and metabolic remodeling of the tumor immune microenvironment following neoadjuvant chemotherapy

**Figure 6:**
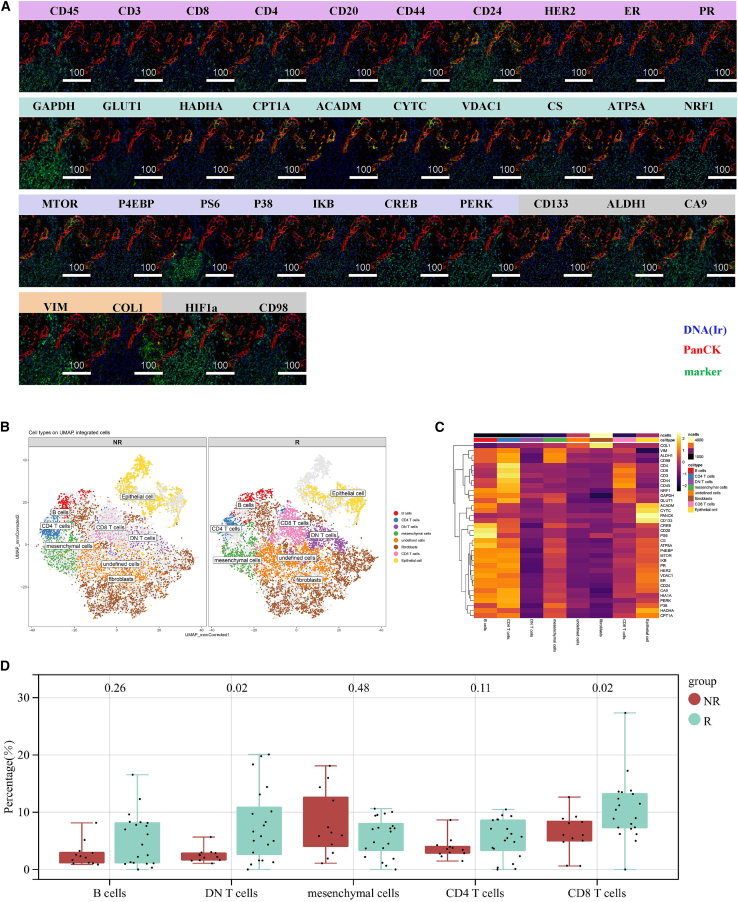
Spatial and cellular profiling of tumor tissues by IMC (A) Representative IMC images showing spatial protein expression patterns in breast tumor tissues (white scale bars, 100 μm). (B) UMAP visualization of single-cell clustering based on protein expression profiles. (C) Heatmap displaying the mean expression levels of key markers across identified cell phenotypes. Color bars range from purple to yellow, with yellow indicating higher expression levels. (D) Boxplots comparing the proportions of non-malignant immune cells between R and NR groups. Statistical significance was determined by two-sided Wilcoxon rank-sum test with Benjamini-Hochberg FDR correction. *n* = 20 responders, 12 non-responders.

**Figure 7:**
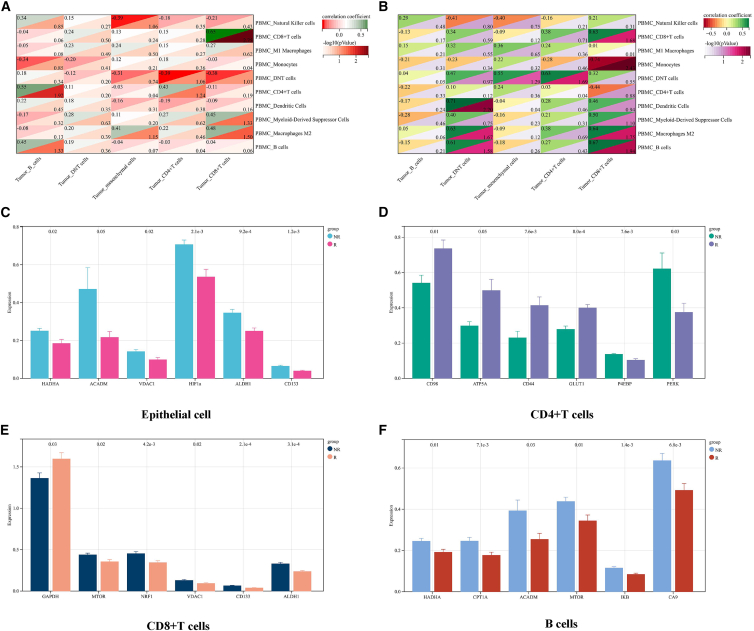
Metabolic characteristics of tumor-infiltrating immune cells in responder patients (A and B) Correlation analyses between tumor-infiltrating non-malignant immune cells and PBMC immune cell fractions before (A) and after (B) neoadjuvant chemotherapy in responder patients. (C) Bar plot showing differential expression of metabolic and stemness-related markers in tumor epithelial cells. (D–F) Bar plots illustrating metabolic differences in tumor-infiltrating CD4^+^ T cells (D), CD8^+^ T cells (E), and B cells (F) between R and NR groups. Statistical significance was determined by two-sided Wilcoxon rank-sum test with Benjamini-Hochberg FDR correction. *n* = 20 responders, 12 non-responders.

To elucidate the mechanisms underlying differential responses to neoadjuvant chemotherapy and to explore the link between intratumoral and peripheral immune states, we performed imaging mass cytometry (IMC) to profile the spatial distribution and metabolic features of tumor-infiltrating cells (Figure 6A). UMAP-based dimensionality reduction and clustering analysis at the single-cell level enabled the definition of distinct cellular populations within tumor tissues (Figure 6B). Heatmap visualization further illustrated the expression of key markers across these cell types (Figure 6C). Comparison of cellular composition revealed that tumors from R patients contained higher proportions of B cells, DNT cells, CD4^+^ T cells, and CD8^+^ T cells compared with NR tumors. Among these, DNT and CD8^+^ T cells showed statistically significant differences (Figure 6D). Correlation analysis between intratumoral non-malignant immune cells and PBMCs demonstrated that, prior to treatment, the frequencies of B cells (r = 0.45), CD4^+^ T cells (r = 0.43), and CD8^+^ T cells (r = 0.65) were positively correlated in R patients, whereas after treatment, only CD8^+^ T cells maintained this correlation (r = 0.63) (Figures 7A and 7B). In contrast, such correlations were not evident in NR patients (Figures S2A and S2B). IMC-based metabolic profiling revealed that NR tumors exhibited a pronounced hypoxic signature, characterized by elevated expression of HIF1α. Consistent with a hypoxia-driven metabolic adaptation, key enzymes involved in fatty acid oxidation (FAO), including HADHA and ACADM, were markedly upregulated in NR tumor epithelial cells, indicating a reliance on FAO for energy production. The high expression of the mitochondrial porin VDAC1 further reflected elevated metabolic stress and mitochondrial activity. Notably, NR tumor cells displayed increased expression of stemness-associated markers ALDH1 and CD133, suggesting enhanced self-renewal capacity, therapy resistance, and poor prognosis. Collectively, NR tumors manifested a malignant phenotype characterized by hypoxia-induced FAO dependency and enriched stem-like features, which likely contribute to the establishment of an immunosuppressive microenvironment through metabolic competition and inhibitory signaling (Figures 7C and S2C). Infiltrating CD4^+^ T cells in NR tumors displayed a unique “high-stress, low-efficiency” metabolic phenotype. The elevated expression of the endoplasmic reticulum stress sensor PERK indicated severe cellular stress, while reduced ATP5A levels reflected impaired mitochondrial oxidative phosphorylation and insufficient energy supply. Despite showing partial activation signals (e.g., upregulated P4EBP), NR CD4^+^ T cells exhibited restricted expression of the amino acid transporter CD98, glucose transporter GLUT1, and activation/memory marker CD44, collectively depicting a metabolically exhausted and functionally limited state (Figures 7D and S2D). These dysfunctional CD4^+^ T cells likely fail to provide adequate helper signals for cytotoxic and humoral immune responses, contributing to chemotherapy resistance. NR tumors also harbored metabolically and functionally dysregulated CD8^+^ T cells. The upregulation of mitochondrial stress–related markers NRF1 and VDAC1, together with stemness marker CD133, suggested that NR CD8^+^ T cells were transitioning toward an exhausted pre-dysfunctional state. Although mTOR activity was elevated, indicating a metabolically active phenotype, the loss of glycolytic enzyme GAPDH expression suggested ineffective metabolic reprogramming to support effector functions. Consequently, these cells exhibited a “high-stress, high-exhaustion-potential, low-efficiency” state, characterized by activation without effective cytotoxicity, thereby facilitating immune evasion and therapeutic resistance (Figures 7E and S2E). B cells infiltrating NR tumors demonstrated a metabolic shift toward lipid utilization, as evidenced by significantly elevated expression of FAO enzymes HADHA, CPT1A, and ACADM. Concurrent upregulation of hypoxia marker CA9 and NF-kB inhibitory protein IKB suggested that these B cells resided in a hypoxic, activation-suppressed microenvironment. Although mTOR signaling appeared activated, this activation was likely ineffective or misdirected. Together, these features define a “hypoxic, metabolically reprogrammed, functionally impaired” phenotype consistent with regulatory or immunosuppressive B cells (Figures 7F and S2F).

Overall, NR tumors were characterized by a hypoxia-driven, FAO-dependent metabolic landscape and an immunosuppressive microenvironment composed of metabolically stressed and functionally exhausted T and B cells. This systemic dysfunction of tumor-infiltrating lymphocytes, coupled with metabolically aggressive tumor epithelial cells, likely underlies the reduced chemosensitivity observed in non-responding patients. “These spatial and metabolic findings were further corroborated by transcriptomic analyses, which revealed parallel immune activation signatures in responder tumors.”

## Discussion

Breast cancer is one of the most common cancers among women worldwide. Common treatment modalities include surgery, radiotherapy, and chemotherapy.^9^ Immune therapy has emerged as a breakthrough in cancer treatment in recent years, by either alleviating immune suppression or directly activating immune cells to exert anti-tumor effects.^10^ Immune cells play a multifaceted and complex role in tumors. They not only participate in tumor immune surveillance, recognizing and eliminating cancer cells, but may also contribute to tumor progression and immune evasion. The role of immune cells in the tumor microenvironment is closely related to their type, subtypes, and functional state.^11^ Although pre-surgical tumor biopsy provides insights into various tumor characteristics, including immune features, substantial heterogeneity exists within different regions of the tumor. It has been shown in multiple cancers that tumor-infiltrating immune cells correlate with peripheral blood immune cells, and detecting peripheral blood immune cells can provide an overall evaluation of tumor-infiltrating immune cells.^12^ Neoadjuvant chemotherapy (NAC) has traditionally been regarded as a cytotoxic intervention targeting rapidly proliferating tumor cells. However, accumulating evidence indicates that its therapeutic efficacy is closely linked to the capacity of the host immune system to mount and sustain effective antitumor responses. In this study, by integrating CyTOF-based immune phenotyping, metabolic profiling, transcriptomics, and spatial imaging, we propose an immune-metabolic framework in which chemotherapy response in TNBC is closely associated not merely with immune cell abundance, but with the coordinated metabolic and functional fitness of systemic and intratumoral lymphocyte populations.

Responders and non-responders diverge along distinct immune-metabolic trajectories following NAC. In responders, chemotherapy was associated with an expansion of effector memory CD4^+^ and CD8^+^ T cells as well as activated and mature B-cell populations exhibiting enhanced glycolytic activity and oxidative phosphorylation. Such metabolic features are consistent with sustained cytokine production, preserved effector function, and improved cellular persistence, characteristics commonly linked to effective antitumor immune responses. In contrast, non-responders were enriched for terminally differentiated or senescent immune subsets characterized by hypoxia-associated FAO, mitochondrial stress, and reduced metabolic flexibility. Previous studies have associated this metabolic program with immune dysfunction and exhaustion, suggesting that metabolic maladaptation, rather than immune cell scarcity, may underlie resistance to chemotherapy. Notably, these immune-metabolic differences were not confined to the peripheral circulation. Spatial and transcriptomic analyses revealed that tumors from responders were enriched for immune activation pathways, including T cell receptor signaling, B cell receptor signaling, and cytokine-cytokine receptor interactions, accompanied by increased infiltration of T and B cells. In contrast, non-responder tumors exhibited a hypoxia-dominated, FAO-biased metabolic landscape, with elevated expression of HIF1α, CPT1A, and other FAO-related enzymes in both tumor epithelial cells and infiltrating immune cells. The convergence of tumor hypoxia and immune metabolic stress may create a metabolically restrictive microenvironment that is unfavorable for effective antitumor immunity.

A key observation of this study is the coordinated association between peripheral and intratumoral immune states in responders, which was largely absent in non-responders. The positive correlation between circulating immune subsets and tumor-infiltrating lymphocytes suggests that effective systemic immune priming may be linked to enhanced immune surveillance within the tumor following NAC. Conversely, the decoupling observed in non-responders implies that circulating immune cells may fail to infiltrate or function efficiently within the tumor microenvironment, potentially due to metabolic incompatibility imposed by hypoxia, nutrient deprivation, or competition. This peripheral-intratumoral immune-metabolic axis provides a systems-level framework to explain how similar immune cell populations can be associated with divergent clinical outcomes.

From a translational standpoint, these findings suggest that immune-metabolic profiling of peripheral blood could serve as a minimally invasive approach to stratify patients according to their likelihood of responding to NAC. Patients enriched for metabolically active effector memory T cells and mature B cells may represent favorable candidates for standard chemotherapy, whereas those exhibiting FAO-biased, metabolically stressed immune profiles may benefit from alternative or combinatorial therapeutic strategies, including immunotherapy or metabolic modulation. Early identification of such immune-metabolic states may facilitate treatment adaptation prior to surgery and improve clinical decision-making.

In summary, this study advances a conceptual immune-metabolic framework in which NAC responsiveness in TNBC is associated with the metabolic adaptability and functional state of immune cells rather than immune cell quantity alone. By integrating systemic and spatial immune profiling, our findings generate testable hypotheses regarding the role of immune-metabolic fitness in chemotherapy response and resistance. Future functional studies will be required to validate these proposed mechanisms and to determine whether therapeutic modulation of immune metabolism can enhance treatment efficacy.

### Limitations of the study

This study has several limitations. First, the cohort size was relatively small, and all patients were enrolled within the past two years; thus, long-term outcomes such as recurrence-free and overall survival are not yet available. A prospective study with a 5-year follow-up has been initiated to validate the prognostic value of the identified immune-metabolic features. Second, while this work focused on peripheral immune-metabolic alterations as a non-invasive biomarker platform, functional validation using single-cell transcriptomics and metabolic flux assays is planned to elucidate underlying regulatory mechanisms. Finally, future studies will integrate stromal and epithelial components into spatial and multi-omics analyses to comprehensively model tumor microenvironment interactions and their impact on chemotherapeutic response.

All patients in this study received standard AC-T chemotherapy, which reflects the current standard-of-care for early-stage TNBC in China, where pembrolizumab is not yet routinely available.

## Resource availability

### Lead contact

Further information and requests for resources and reagents should be directed to and will be fulfilled by the lead contact, He Ren (202201491@hrbmu.edu.cn).

### Materials availability

This study did not generate new unique reagents.

### Data and code availability

• The datasets generated in this study have been deposited in the National Genomics Data Center (NGDC) under accession number OMIX015531 and are publicly available.
• All other data supporting the findings of this study are available from the lead contact upon reasonable request.
• The code used for data processing, clustering, and visualization is available from the lead contact upon reasonable request.

## Acknowledgments

This work was supported by grants from 10.13039/501100001809National Natural Science Foundation of China (82073146 to Zhigao Li, 82503605 to Rrunze Shi), the Cultivation Project of the Joint Fund of the 10.13039/501100005046Heilongjiang Provincial Natural Science Foundation (PL2025H176 to He Ren), the Heilongjiang Provincial General Postdoctoral Funding Program (LBH-Z25229 to He Ren), and the 10.13039/100010722Harbin Medical University Youth Fund (2023-KYYWF-0202 to Wenzheng Wang).

## Author contributions

R.S.: investigation and resources; Y.P.: validation, formal analysis, and data curation; C.C. and K.L.: data curation; Wenjing Wang: investigation; Y.S.: methodology; Z.L.: writing – review and editing and project administration; H.R.: writing – review and editing and writing – original draft; Wenzheng Wang: writing – review and editing and writing – original draft.

## Declaration of interests

The authors declare no competing interests.

## STAR★Methods

### Key resources table

**Table:** 

REAGENT or RESOURCE	SOURCE	IDENTIFIER
**Antibodies**		

Anti-human GAPDH (clone EPR16891)	Abcam	Cat# ab199553
Anti-human ER (clone EPR4097)	Abcam	Cat# ab167610
Anti-human HADHA (clone EPR17940)	Abcam	Cat# ab231169
Anti-human CD8 (clone RM1129)	Abcam	Cat# ab316779
Anti-human CD20 (clone SP32)	Abcam	Cat# ab236434
Anti-human CD4 (clone EPR6855)	Abcam	Cat# ab181724
Anti-human CPT1A (clone 8F6AE9)	Abcam	Cat# ab128568
Anti-human ACADM (clone 3B7BH7)	Abcam	Cat# ab110296
Anti-human Cytochrome *c* (clone EPR1327)	Abcam	Cat# ab218312
Anti-human VDAC1 (clone EPR27552-6)	Abcam	Cat# ab306582
Anti-human GLUT1 (clone EPR3915)	Abcam	Cat# AB196357
Anti-human CD3 (clone CD3-12)	Abcam	Cat# ab11089
Anti-human pan-CK (clone C-11)	Abcam	Cat# ab264485
Anti-human CD133 (clone RM1002)	Abcam	Cat# ab282745
Anti-human CD44 (clone RM1084)	Abcam	Cat# ab316124
Anti-human CD24 (clone EPR19925)	Abcam	Cat# ab199140
Anti-human CD45 (clone MEM-28)	Abcam	Cat# ab8216
Anti-human NRF1 (clone EPR5554(N))	Abcam	Cat# ab221792
Anti-human Collagen I (clone RM1131)	Abcam	Cat# ab316223
Anti-human ALDH1 (clone EP1933Y)	Abcam	Cat# ab215996
Anti-human Vimentin (clone EPR3776)	Abcam	Cat# ab193555
Anti-human CD98 (clone EPR27110-42)	Abcam	Cat# ab307588
Anti-human HIF-1α (clone EPR3658)	Abcam	Cat# ab214440
Anti-human mTOR (clone Y391)	Abcam	Cat# ab218525
Anti-human p-4E-BP1 (clone EPR2169Y)	Abcam	Cat# ab247374
Anti-human CS (clone EPR8067)	Abcam	Cat# ab233838
Anti-human p38 (clone EPR16587)	Abcam	Cat# ab236527
Anti-human phospho-S6 (clone Y179)	Abcam	Cat# ab247235
Anti-human IKB-α (clone E130)	Abcam	Cat# ab215972
Anti-human CREB (clone E113)	Abcam	Cat# ab220798
Anti-human ATP5A (clone 7H10BD4F9)	Abcam	Cat# ab110273
Anti-human *p*-ERK (clone pT202/pY204.22A)	Santa Cruz	Cat# sc-136521
Anti-human CA9 (clone EPR23055-5)	Abcam	Cat# ab270401
Anti-human HER2 (clone EPR19547-12)	Abcam	Cat# ab222482
Anti-human PR (clone YR85)	Abcam	Cat# ab206926
Anti-human CD45 (clone HI30)	Fluidigm	Cat# 3089003B
Anti-human PKM2 (clone EPR10138(B))	Abcam	Cat# ab150377
Anti-human IFN-γ (clone EPR23991-53)	Abcam	Cat# ab267369
Anti-human CD45RA (clone HI100)	Fluidigm	Cat# 3143006B
Anti-human GLUT1 (clone SP168)	Abcam	Cat# SLC2A1
Anti-human CD20 (clone 2H7)	POLARIS	Cat# H10612097
Anti-human CD8 (clone RPA-T8)	Fluidigm	Cat# 3146001B
Anti-human CD45RO (clone UCHL1)	POLARIS	Cat# H21612105
Anti-human mTOR (clone EPR427(N))	Abcam	Cat# ab137133
Anti-human CD25 (clone 2A3)	Fluidigm	Cat# 3149010B
Anti-human SDH (clone EPR9043(B))	Abcam	Cat# ab137040
Anti-human CD11b (clone LM2)	Biolegend	Cat# 393102
Anti-human TNF-α (clone Mab11)	Fluidigm	Cat# 3146010B
Anti-human CPT1A (clone EPR21843-71-1C)	Abcam	Cat# ab220789
Anti-human LDH (clone EP1565Y)	Abcam	Cat# ab208366
Anti-human PD-1 (clone EH12.2H7)	POLARIS	Cat# H15811115
Anti-human PGC1A (clone 4A8)	Abcam	Cat# ab77210
Anti-human IDH (clone EPR21002)	Abcam	Cat# ab242078
Anti-human CD86 (clone IT2.2)	POLARIS	Cat# H26712TBD
Anti-human CD19 (clone HIB19)	POLARIS	Cat# H09212119
Anti-human CD206 (clone 15–2)	POLARIS	Cat# H11321TBD
Anti-human CD11c (clone Bu15)	POLARIS	Cat# H03112132
Anti-human IL-10 (clone EPR1114)	Abcam	Cat# ab133575
Anti-human CD98 (clone MEM-108)	Biolegend	Cat# 315602
Anti-human CD36 (clone EPR22509-40)	Abcam	Cat# ab255331
Anti-human CD56 (clone NCAM16.2)	POLARIS	Cat# H23414131
Anti-human CCR7 (clone G043H7)	POLARIS	Cat# H09813132
Anti-human CD4 (clone RPA-T4)	Biolegend	Cat# 300501
Anti-human CD14 (clone M5E2)	Biolegend	Cat# 301801
Anti-human IL-17 (clone QA18A46)	Biolegend	Cat# 385902
Anti-human CD57 (clone TB01)	Abcam	Cat# ab33273
Anti-human HLA-DR (clone LN3)	Fluidigm	Cat# 3174025D
Anti-human CD68 (clone FA-11)	POLARIS	Cat# H24941133
Anti-human CD3 (clone UCHT-1)	POLARIS	Cat# H16712189

**Chemicals, peptides, and recombinant proteins**		

Lymphoprep	Stemcell	Cat# 07851
SepMate-50	Stemcell	Cat# 85450
TCEP	Thermo Scientific	Cat# 77720
FixI	Standard Biotools	Cat# S00115
Perm-S	Standard Biotools	Cat# 201066
Cell-ID Intercalator-Ir	Standard Biotools	Cat# S00093
EQ Four Element Calibration Beads	Standard Biotools	Cat# 201078
20-Plex Pd Barcoding Kit	Standard Biotools	Cat# S00114
Maxpar X8 Multi-Metal Labeling Kit	Standard Biotools	Cat# S00009
Red blood cell lysis solution	Solarbio	Cat# R1010
BSA	Solarbio	Cat# A8020
DPBS	Corning	Cat# 21-031-CVR
EDTA anticoagulant tubes	BD	Cat# 367525
Amicon Ultra 3 kDa filter	Merck	Cat# UFC500396
Amicon Ultra 50 kDa filter	Merck	Cat# UFC5050BK

**Deposited data**		

Raw sequencing data	National Genomics Data Center (NGDC)	OMIX015531

**Software and algorithms**		

CyTOF 7.1	Standard Biotools	https://www.standardbiotools.com/cytof-software
Cytobank	Cytobank Inc.	https://www.cytobank.org
R (v4.3.0)	R Foundation	https://www.r-project.org
cytofkit	Bioconductor	https://bioconductor.org/packages/cytofkit
DESeq2 (v1.40.2)	Bioconductor	https://bioconductor.org/packages/DESeq2
fastp (v0.23.1)	GitHub	https://github.com/OpenGene/fastp
HISAT2 (v2.2.1)	Daehwan Kim Lab	https://daehwankimlab.github.io/hisat2/
imcRtools	Bioconductor	https://bioconductor.org/packages/imcRtools
histoCAT	GitHub	https://github.com/BodenmillerGroup/histoCAT
Sangerbox	Sangerbox	http://www.sangerbox.com

### Experimental model and study participant details

#### Human participants

This study was approved by the Ethics Committee of Harbin Medical University Cancer Hospital (KY2023-66, approved on October 10, 2023) and conducted in accordance with the Declaration of Helsinki, all participants were Homo sapiens. A total of 32 triple-negative breast cancer (TNBC) patients undergoing neoadjuvant chemotherapy (NAC) and 20 healthy female controls were enrolled. Patients were recruited consecutively from Harbin Medical University Cancer Hospital between October 2023 and August 2024. Inclusion criteria were: large tumor size (T3 or higher) and lymph node metastasis. Exclusion criteria included history of severe thrombocytopenia, significant hepatic dysfunction, pre-existing severe pulmonary diseases, current respiratory complications, or hypersensitivity to chemotherapy drugs. The mean age of patients was 54.3 years (range: 39–75), and the control group had a mean age of 56.3 years (*p* = 0.227, independent *t* test). All participants provided written informed consent. According to RECIST 1.1 criteria evaluated after completion of chemotherapy, patients with ≥30% tumor reduction or complete response were classified as responders (R, *n* = 20), and those with <30% reduction or stable disease as non-responders (NR, *n* = 12). All participants in this study were female, as TNBC predominantly occurs in women. Therefore, the influence of sex or gender on the results could not be assessed.

#### Cell lines

No cell lines were used in this study.

### Method details

#### Neoadjuvant chemotherapy regimen

Patients received AC-T chemotherapy: four cycles of AC (doxorubicin and cyclophosphamide) administered every 21 days, followed by four sequential cycles of T (paclitaxel). Blood samples were collected one week prior to initiation of chemotherapy and one week after completion of the final cycle. Tumor tissues were collected at the time of surgery following NAC.

#### Antibody metal labeling

Antibodies were labeled using the Maxpar X8 Multi-Metal Labeling Kit (Standard Biotools) following the manufacturer’s protocol. Briefly, 95 μL of L-buffer was added to the X8 polymer tube and mixed until the polymer dissolved, followed by addition of 5 μL of 50 mM lanthanide metal solution and incubation at 37°C for 45 min. Meanwhile, 100 μg of antibody was transferred to a 50 kDa filter column, adjusted to 500 μL with R-buffer, and centrifuged at 12,000g for 10 min. TCEP was diluted to 4 mM in R-buffer, and 100 μL was added to the antibody-containing filter column and incubated at 37°C for 30 min. The metal-polymer mixture was transferred to a 3 kDa filter column and centrifuged at 12,000g for 30 min. The labeled antibody was washed with C-buffer and W-buffer, collected in antibody storage buffer, and stored at 4°C.

#### Peripheral blood mononuclear cell (PBMC) isolation and preparation

Whole blood collected in EDTA tubes was centrifuged at 800g for 10 min to separate plasma. PBMCs were isolated by density gradient centrifugation using Lymphoprep (Stemcell) and SepMate tubes. Gradient centrifugation was performed at 1,200g for 15 min (acceleration 3, deceleration 1). The PBMC layer was collected, washed with DPBS containing 5% FBS, and centrifuged at 400g for 8 min. Red blood cells were lysed using red blood cell lysis solution (Solarbio). Live cells were labeled with 0.01% cisplatin for 2 min at room temperature. Cells were fixed with FixI (Standard Biotools) for 10 min, washed, and stored at −80°C in cell storage solution.

#### PBMC barcoding and antibody staining

PBMCs were barcoded using the 20-Plex Pd Barcoding Kit (Standard Biotools) according to the manufacturer’s instructions. Barcoded samples were combined, stained with surface antibodies for 30 min at room temperature, fixed with FixI, and permeabilized with Perm-S (Standard Biotools). Intracellular antibodies were added and incubated for 30 min at room temperature. Cells were stained overnight with 0.1% Cell-ID Intercalator-Ir (Standard Biotools) at 4°C. Before acquisition, samples were washed with pure water, resuspended in cell collection buffer containing 10% EQ Four Element Calibration Beads (Standard Biotools), and filtered through a 40 μm mesh.

#### CyTOF data acquisition and analysis

Data were acquired on a Helios mass cytometer (Standard Biotools). Raw FCS files were normalized using CyTOF 7.1 software and debarcoded to separate individual samples. Processed data were uploaded to Cytobank (https://www.cytobank.org) for cleaning: EQ beads, debris, aggregates, and dead cells (cisplatin-positive) were removed, and CD45^+^ immune cells were selected for analysis. Clustering was performed using PhenoGraph (k = 35) and FlowSOM (xdim = 40, ydim = 40, meta-clustering k = 15) in R via the cytofkit package. Dimensionality reduction was performed using t-SNE (max_iter = 2500, perplexity = 35) on a random subset of 10,000 cells per sample. Immune subsets were annotated based on canonical lineage, activation, and differentiation markers as listed in Table S1.

#### Imaging Mass Cytometry (IMC)

Formalin-fixed paraffin-embedded (FFPE) tumor sections were baked at 60°C for 2 hours, deparaffinized in xylene, and rehydrated through a graded ethanol series. Heat-induced epitope retrieval was performed using pH 9 buffer at 96°C for 30 min. Sections were blocked with 3% BSA in Maxpar PBS for 45 min and incubated overnight at 4°C with a metal-tagged antibody cocktail (antibodies listed in Table S2) prepared in Maxpar PBS containing 0.5% BSA. After washing, sections were stained with Cell-ID Intercalator-Ir (1:400) for 30 min, washed with Maxpar Water, and air-dried. Data were acquired on a Hyperion Imaging System. Raw MCD files were normalized and segmented using imcRtools and histoCAT pipelines. Single-cell data were analyzed in R (v4.3.0) for cell phenotyping, neighborhood enrichment, and spatial mapping.

#### Transcriptome sequencing and analysis

Total RNA was extracted from tumor tissue samples. mRNA was enriched using poly-T oligo-attached magnetic beads. Sequencing libraries were constructed and sequenced on an Illumina platform by Novogene Co., Ltd. Raw FASTQ reads were processed with fastp (v0.23.1) to remove adapter contamination and low-quality bases (Phred <5). Clean reads were aligned to the human reference genome (GRCh38) using HISAT2 (v2.2.1), and transcript abundance was quantified as FPKM values. Differential expression analysis between R and NR groups was performed using DESeq2 (v1.40.2). Genes with absolute fold change ≥2 and adjusted *p*-value (Benjamini-Hochberg FDR) < 0.05 were considered differentially expressed. Immune cell composition was estimated using xCell.

### Quantification and statistical analysis

All statistical analyses were performed in R (v4.3.0). For transcriptomic data, differential expression was assessed using DESeq2 with Benjamini-Hochberg FDR correction; significance was defined as adjusted *p* < 0.05. For CyTOF and IMC data, pairwise comparisons were evaluated using two-sided t-tests or Wilcoxon rank-sum tests, with *p*-values subjected to Benjamini-Hochberg FDR correction; significance was defined as FDR <0.05. Correlation analyses were performed using Pearson correlation. Detailed statistical parameters, including exact *n* values (representing number of patients), test statistics, and *p*-values, are reported in the figure legends.

### Additional resources

Clinical trial registration: Not applicable. No new websites or forums were created for this study.
