# Supplementary material for: Immune-metabolic profiling of triple-negative breast cancer during neoadjuvant chemotherapy
Source: iScience. 2026 Apr 22;29(6):115845. doi: 10.1016/j.isci.2026.115845 (PMC13156682; doi:10.1016/j.isci.2026.115845)
Supplement: Document S1. Figures S1 and S2 and Tables S1 and S2 [file mmc1.pdf]

iScience, Volume *29*

Supplemental information

Immune-metabolic profiling of triple-negative breast cancer during neoadjuvant chemotherapy

Runze Shi, Yuheng Pang, Chen Chen, Kexin Liu, Wenjing Wang, Yuefeng Shang, Zhigao Li, He Ren, and Wenzheng Wang

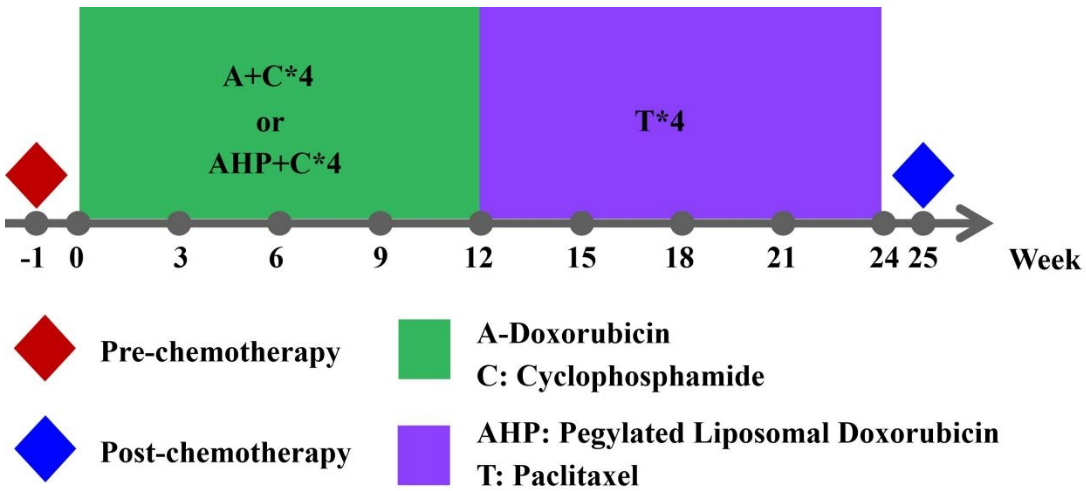

Supplementary Figure 1. Schematic timeline of PBMC sampling during neoadjuvant chemotherapy.

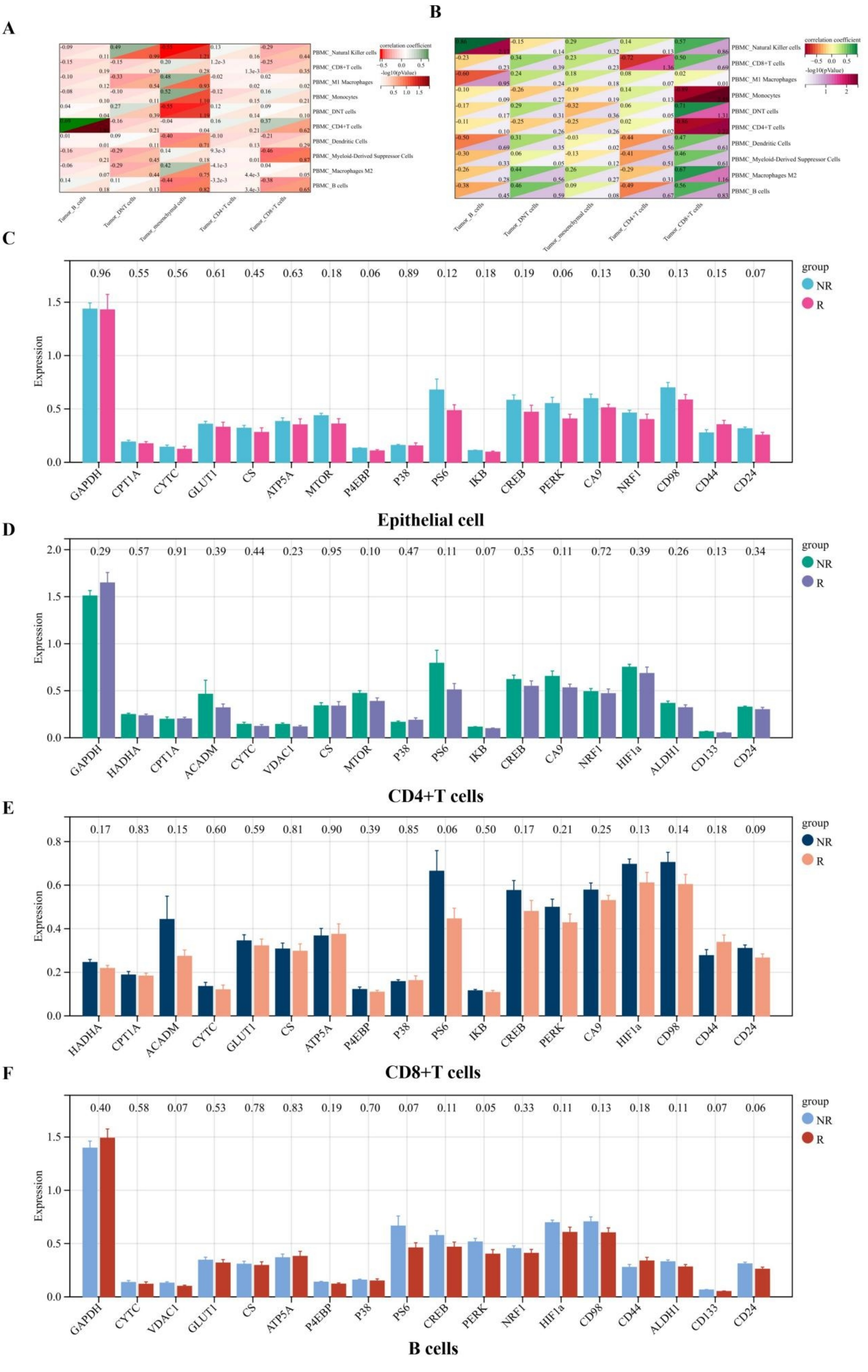
A
B
C
D
E
F
correlation coefficient
-log10(pValue)
PBMC_Natural Killer cells
PBMC_CD8+T cells
PBMC_M1 Macrophages
PBMC_Monocytes
PBMC_DNT cells
PBMC_CD4+T cells
PBMC_Dendritic Cells
PBMC_Myeloid-Derived Suppressor Cells
PBMC_Macrophages M2
PBMC_B cells
Tumor_B cells
Tumor_DNT cells
Tumor_mesenchymal cells
Tumor_CD4+T cells
Tumor_CD8+T cells
group
NR
R
Expression
0.96 0.55 0.56 0.61 0.45 0.63 0.18 0.06 0.89 0.12 0.18 0.19 0.06 0.13 0.30 0.13 0.15 0.07
GAPDH CPT1A CYTC GLUT1 CS ATP5A MTOR P4EBP P38 PS6 IKB CREB PERK CA9 NRF1 CD98 CD44 CD24
Epithelial cell
0.29 0.57 0.91 0.39 0.44 0.23 0.95 0.10 0.47 0.11 0.07 0.35 0.11 0.72 0.39 0.26 0.13 0.34
GAPDH HADHA CPT1A ACADM CYTC VDAC1 CS MTOR P38 PS6 IKB CREB CA9 NRF1 HIF1a ALDH1 CD133 CD24
CD4+T cells
0.17 0.83 0.15 0.60 0.59 0.81 0.90 0.39 0.85 0.06 0.50 0.17 0.21 0.25 0.13 0.14 0.18 0.09
HADHA CPT1A ACADM CYTC GLUT1 CS ATP5A P4EBP P38 PS6 IKB CREB PERK CA9 HIF1a CD98 CD44 CD24
CD8+T cells
0.40 0.58 0.07 0.53 0.78 0.83 0.19 0.70 0.07 0.11 0.05 0.33 0.11 0.13 0.18 0.11 0.07 0.06
GAPDH CYTC VDAC1 GLUT1 CS ATP5A P4EBP P38 PS6 CREB PERK NRF1 HIF1a CD98 CD44 ALDH1 CD133 CD24
B cells

Supplementary Figure 2. Metabolic characteristics of tumor-infiltrating immune cells in non-responder (NR) patients.

(A–B) Correlation analyses between tumor-infiltrating non-malignant immune cells and PBMC immune cell fractions before (A) and after (B) neoadjuvant chemotherapy in non-responder patients. (C) Bar plot showing differential metabolic marker expression in tumor epithelial cells. (D–F) Bar plots illustrating metabolic alterations in tumor-infiltrating $CD4^+$ T cells (D), $CD8^+$ T cells (E), and B cells (F) . Statistical significance was determined by two-sided Wilcoxon rank-sum test with Benjamini-Hochberg FDR correction. n = 20 responders, 12 non-responders.

Supplementary Table 1：Mass cytometry antibodies panel design.

Antigen	Mass	Antibody clone	Source
CD45	89	HI30	Fluidigm
PKM2	141	EPR10138(B)	abcam
IFN-γ	142	EPR23991-53	abcam
CD45RA	143	HI100	Fluidigm
GLUT1	145	SP168	abcam
CD20	144	2H7	POLARIS
CD8	146	RPA-T8	Fluidigm
CD45RO	147	UCHL1	POLARIS
mTOR	148	EPR427(N)	abcam
CD25	149	2A3	Fluidigm
SDH	150	EPR9043(B)	abcam
CD11B	151	LM2	Biolegned
TNFa	152	Mab11	Fluidigm
CPT1A	153	EPR21843-71-1C	abcam
LDH	154	EP1565Y	abcam
PD-1	155	EH12.2H7	POLARIS
PGC1A	156	4A8	abcam
IDH	158	EPR21002	abcam
CD86	159	IT2.2	POLARIS
CD19	160	HIB19	POLARIS
CD206	161	15-2	POLARIS
CD11C	162	Bu15	POLARIS
IL_10	163	EPR-1114	abcam
CD98	164	MEM-108	Biolegned
CD36	165	EPR22509-40	abcam
CD56	166	NCAM16.2	POLARIS
CCR7	167	G043H7	POLARIS
CD4	168	RPA-T4	Biolegned
CD14	169	M5E2	Biolegned
IL-17	170	QA18A46	Biolegned
CD57	172	TB01	abcam
HLA-DR	174	LN3	Fluidigm
CD68	176	FA-11	POLARIS
CD3	209	UCHT-1	POLARIS

Supplementary Table 2：Image Mass cytometry antibodies panel design.

Antigen	Mass	Antibody clone	Source
GAPDH	141	EPR16891	abcam
ER	142	EPR4097	abcam
HADHA	143	EPR17940	abcam
CD8	144	RM1129	abcam
CD20(B)	145	SP32	abcam
CD4	146	EPR6855	abcam
CPT1A	147	8F6AE9	abcam
ACADM	148	3B7BH7	abcam
CYTC	149	EPR1327	abcam
VDAC1	150	EPR27552-6	abcam
GLUT1	151	EPR3915	abcam
CD3	152	CD3-12	abcam
pan-CK	153	C-11	abcam
CD133	154	RM1002	abcam
CD44	155	RM1084	abcam
CD24	156	EPR19925	abcam
CD45	158	MEM-28	abcam
NRF1	159	EPR5554(N)	abcam
COL1	160	RM1131	abcam
ALDH1	161	EP1933Y	abcam
VIM	162	EPR3776	abcam
CD98	163	EPR27110-42	abcam
HIF1a	164	EPR3658	abcam
MTOR	165	Y391	abcam
p4ebp	166	EPR2169Y	abcam
CS	167	EPR8067	abcam
p38	168	EPR16587	abcam
(phospho S6)	169	Y179	abcam
IKB	170	E130	abcam
CREB	171	E113	abcam
ATP5A	172	7H10BD4F9	abcam
pERK	173	pT202/pY204.22A	abcam
CA9	174	EPR23055-5	abcam
HER2	175	EPR19547-12	abcam
PR	176	YR85	abcam
